# Supplementary material for: Clostridioides difficile-mucus interactions encompass shifts in gene expression, metabolism, and biofilm formation
Source: mSphere. 2024 Jun 5;9(6):e00081-24. doi: 10.1128/msphere.00081-24 (PMC11332178; doi:10.1128/msphere.00081-24)
Supplement: Supplemental figures — Figures S1-S4. [file msphere.00081-24-s0004.pdf]

SUPPLEMENTAL FIGURES

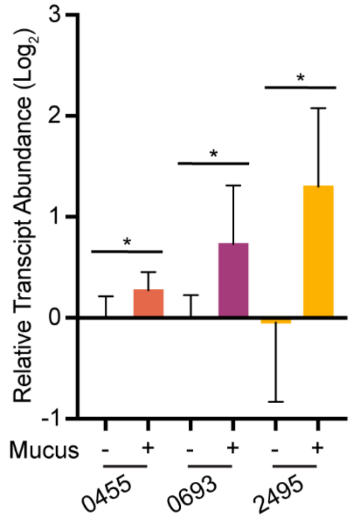

Figure S1. Confirmation of differential expression of genes of interest. Relative transcript abundance determined by qRT-PCR for CDR0455, CDR0693, and CDR0696 in broth cultures recapitulating conditions used for RNA-Seq. Mean expression and standard deviations from n=7 or 8 biological replicates shown, combined from 3 independent experiments. *$p < 0.05$, unpaired, two-tailed t-test.

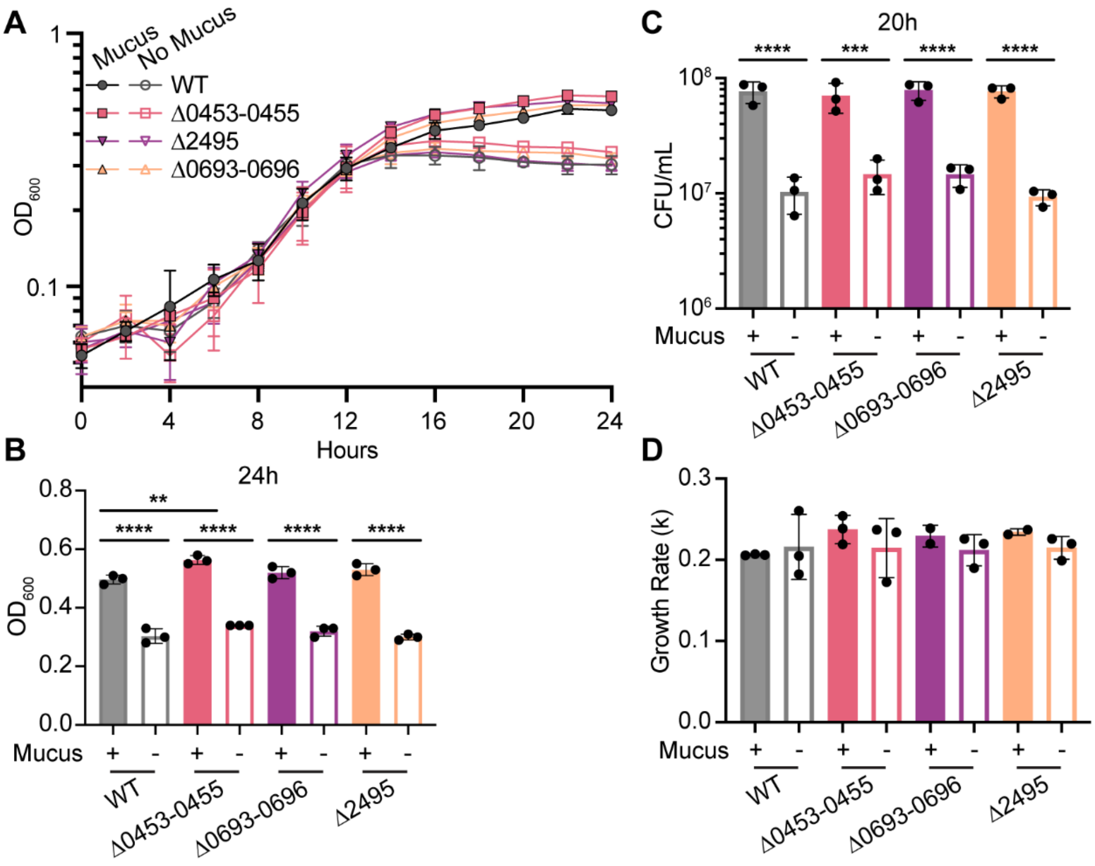

Figure S2. Effects of deletion of genes and operons upregulated with mucus on growth without glucose. (A) Growth curves for mutants and wildtype in CDMM lacking glucose with mucus (filled symbols) and without mucus (open symbols). Data are from one representative experiment, n=3. **(B)** Comparison of OD_{600} values at the final time point. **(C)** Viable cell counts expressed as CFU/mL. **(D)** Growth rates during exponential phase. $^{**}p < 0.01$, $^{***}p < 0.001$, $^{****}p < 0.0001$, one way ANOVA with Sidak's test.

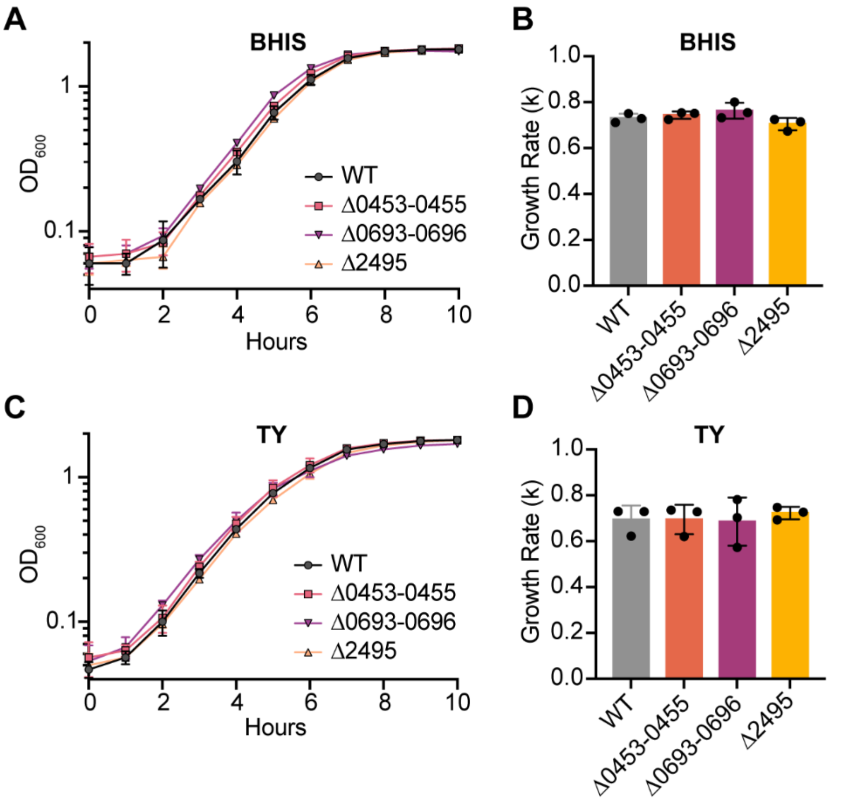

Figure S3. Effects of deletion of genes and operons upregulated with mucus on growth in rich media. (A) Growth curves for mutants and wildtype in 1X BHIS media. **(B)** Growth rates during exponential phase for cultures grown in BHIS. **(C)** Growth curves for mutants and wildtype in 1X TY. Data are from one representative experiment, n=3. **(D)** Growth rates during exponential phase for cultures grown in TY. Data are from one representative experiment, n=3. No differences in growth trends or rates observed.

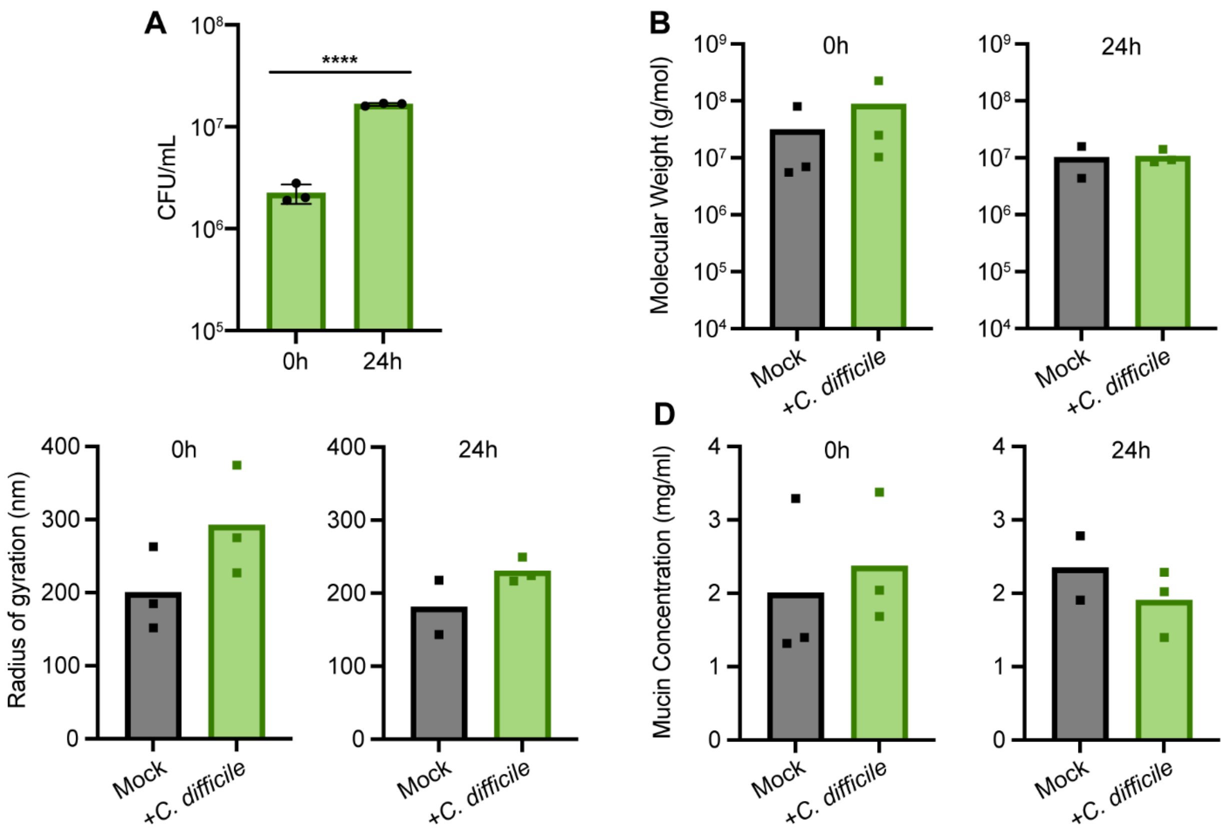

Figure S4. *C. difficile* viability in *ex vivo* mucus and its effect on mucus biochemical properties. (A) Viable cell counts for *C. difficile* in *ex vivo* mucus at time of inoculation and after 24 hours, expressed as CFU/mL. ****p < 0.0001, unpaired, two-tailed t-test. For biochemical analyses, mucins were separated using size exclusion chromatography and detected by multiangle laser light scattering (MALLS), which measured molecular weights **(B)**, radii of gyration **(C)**, or concentration of mucins **(D)**. For MALLS, each sample was run in technical duplicate or triplicate; means for each sample are shown.
