# Supplementary material for: Clostridioides difficile-mucus interactions encompass shifts in gene expression, metabolism, and biofilm formation
Source: mSphere. 2024 Jun 5;9(6):e00081-24. doi: 10.1128/msphere.00081-24 (PMC11332178; doi:10.1128/msphere.00081-24)
Supplement: Supplemental tables — Tables S1 and S2. [file msphere.00081-24-s0005.pdf]

Supplementary Table 1: Differentially expressed genes related to nutrient acquisition or metabolism. L2FC±SE: Log_2 fold change ± standard error.

Fructose and Mannose Metabolism Gene Set (KEGG)			
**Gene**	**Name, Description, & Details**	**L2FC±SE**	**p-adj**
CDR0692	*gutA*; Glucitol/sorbitol PTS system EIIC component; homologous to CD630 *srlA*	1.367±0.104	0.0000
CDR0693	*srlE*; Glucitol/sorbitol PTS system EIIB component; homologous to CD630 *srlEa*	1.498±0.116	0.0000
CDR0694	*srlE'*; Glucitol/sorbitol PTS system EIIB component; homologous to CD630 *srlEb*	1.493±0.094	0.0000
CDR0695	*srlB*; Glucitol/sorbitol PTS system EIIA component	1.493±0.114	0.0000
CDR0696	*gutD*; Sorbitol-6-phosphate 2-dehydrogenase; homologous to CD630 *srlD*	1.176±0.162	0.0000
CDR2904	Mannose PTS system EIIC component	1.285±0.067	0.0000
CDR2175	L-fuculose-phosphate aldolase	1.219±0.190	0.0000
CDR2898	*xylA*; Xylose isomerase	1.040±0.082	0.0000
CDR2452	Fructose PTS system EIIA component	-1.073±0.134	0.0000
CDR2976	Fructose PTS system EIIA component	-1.221±0.229	0.0000
Wood-Ljungdahl Pathway			
**Gene**	**Name, Description, & Details**	**L2FC±SE**	**p-adj**
CDR3174	*hydN1*; electron transport protein	0.441±0.070	0.0000
CDR3175	*hydA*; hydrogenase	0.215±0.066	0.0018
CDR3176	*hydN2*; electron transport protein	0.361±0.075	0.0000
CDR3177	hypothetical protein; homologous to CD630 *fdh*	0.489±0.171	0.0042
CDR3178	*fdhD*; formate dehydrogenase accessory protein	0.214±0.081	0.0115
CDR3179	*fdhF*; formate dehydrogenase	0.503±0.067	0.0000
CDR0648	conserved hypothetical protein; homologous to CD630 *metV*	0.270±0.079	0.0009
CDR0649	putative methylenetetrahydrofolate reductase; homologous to CD630 *metF*	0.270±0.089	0.0034
CDR0643	*cooS*; putative bifunctional carbon monoxide dehydrogenase/acetyl-CoA synthase; homologous to CD630 *acsA*	0.522±0.074	0.0000
CDR0652	putative carbon monoxide dehydrogenase/acetyl-CoA synthase complex, small subunit, homologous to CD630 *acsE*	0.516±0.100	0.0000
CDR0653	putative carbon monoxide dehydrogenase/acetyl-CoA synthase complex, alpha subunit, homologous to CD630 *acsC*	0.853±0.068	0.0000
CDR0654	putative carbon monoxide dehydrogenase/acetyl-CoA synthase complex, methyltransferase subunit, homologous to CD630 *acsE*	0.575±0.064	0.0000
CDR0655	putative carbon monoxide dehydrogenase/acetyl-CoA synthase complex, beta subunit, homologous to CD630 *acsB*	0.460±0.069	0.0000
CDR0111	*ptb;* phosphate butyryltransferase	-0.908±0.078	0.0000
CDR2571	putative propanediol utilization protein; homologous to CD630 *pta*	-0.233±0.069	0.0010
CDR1012	*ackA; acetate kinase*	-0.909±0.058	0.0000
CDR0112	*buk*; butyrate kinase	-0.596±0.064	0.0000
CDR0915	*thlA1*; acetyl-CoA acetyltransferase, homologous to CD630 *thlA*	-0.534±0.077	0.0000
CDR0914	*hbd*; 3-hydroxybutyryl-CoA dehydrogenase	-0.449±0.088	0.0000
CDR0913	*crt2*; 3-hydroxybutyryl-CoA dehydratase	-0.516±0.105	0.0000
CDR0912	*etfA2*; electron transfer flavoprotein alpha-subunit	-0.505±0.079	0.0000
CDR0911	*etfB2*; electron transfer flavoprotein beta-subunit	-0.386±0.083	0.0000
CDR0910	*bcd2*; butyryl-CoA dehydrogenase	-0.248±0.067	0.0003
CDR2800	*adhE*; aldehyde-alcohol dehydrogenase	-0.312±0.060	0.0000
Glycine Cleavage System			

Gene	Name, Description, & Details	L2FC±SE	p-adj
CDR0656	*gcvH*; putative glycine cleavage system H protein	0.458±0.086	0.0000
CDR0650	putative carbon monoxide dehydrogenase/acetyl-CoA synthase complex, dihydrolipoyl dehydrogenase subunit; homologous to CD630 *gcvL*	0.347±0.077	0.0000
CDR1556	*gcvPB*; glycine cleavage system P protein	-0.909±0.077	0.0000
CDR1555	putative bi-functional glycine dehydrogenase/aminomethyl transferase protein, homologous to CD630 *gcvT*	-0.601±0.068	0.0000
CDR2615	*glyA*; putative serine hydroxymethyltransferase	0.366±0.070	0.0000
CDR3082	*sdaB*; L-serine dehydratase	0.707±0.067	0.0000
Proline and Glycine Reductases (Stickland Metabolism)			
**Gene**	**Name, Description, & Details**	**L2FC±SE**	**p-adj**
CDR3097	*prdF*; putative proline racemase	-1.793±0.061	0.0000
CDR3098	conserved hypothetical protein	-2.229±0.064	0.0000
CDR3099	conserved hypothetical protein	-2.180±0.089	0.0000
CDR3100	conserved hypothetical protein	-2.204±0.069	0.0000
CDR3101	*prdB*; proline reductase	-2.245±0.061	0.0000
CDR3103	*prdA*; proline reductase subunit protein	-2.224±0.070	0.0000
CDR3104	*prdR*; sigma-54-dependent transcriptional activator	-0.691±0.083	0.0000
CDR3105	*prdC*; putative electron transfer protein	-1.249±0.083	0.0000
CDR2234	LysR-family regulatory protein	0.556±0.103	0.0000
CDR2235	putative membrane protein	0.421±0.143	0.0036
CDR2236	putative Xaa-Pro dipeptidase	0.258±0.085	0.0034
CDR2237	*grdD*; glycine/sarcosine/betaine reductase complex component C alpha subunit	-3.460±0.070	0.0000
CDR2238	*grdC*; glycine/sarcosine/betaine reductase complex component C beta subunit	-3.306±0.081	0.0000
CDR2239	*grdB*; glycine reductase complex component B gamma subunit	-1.207±0.078	0.0000
CDR2240	*grdA*; glycine/sarcosine/betaine reductase complex component A	-2.292±0.073	0.0000
CDR2241	*grdE*; glycine reductase complex component B alpha and beta subunits	-3.399±0.073	0.0000
CDR2242	*trxA2*; thioredoxin	-3.148±0.101	0.0000
CDR2243	*trxB3*; thioredoxin reductase	-3.155±0.071	0.0000
CDR2244	*grdX*; putative glycine reductase complex component	-2.502±0.085	0.0000

Supplementary Table 2: Differentially expressed genes related to transcriptional regulation or sensing. L2FC±SE: Log_2 fold change ± standard error.

Transcriptional Regulators			
**Gene**	**Name, Description, & Details**	**L2FC±SE**	**p-adj**
CDR0508	TetR-family transcriptional regulator	2.010±0.072	0.0000
CDR1650	putative transcriptional regulator	1.670±0.072	0.0000
CDR1351	putative transcriptional regulator	1.610±0.167	0.0000
CDR1936	GntR-family transcriptional regulator	1.574±0.098	0.0000
CDR1579	TetR-family transcriptional regulator	1.541±0.071	0.0000
CDR0865	GntR-family transcriptional regulator	1.534±0.104	0.0000
CDR1467	MarR-family transcriptional regulator	1.410±0.077	0.0000
CDR0310	TetR putative transcriptional regulator	1.367±0.089	0.0000
CDR1646	TetR-family transcriptional regulator	1.273±0.076	0.0000
CDR2050	putative transcriptional regulator	1.216±0.140	0.0000
CDR0317	ArsR-family transcriptional regulator	1.194±0.087	0.0000
CDR2553	AraC-family transcriptional regulator	1.185±0.085	0.0000
CDR1619	putative transcriptional regulator	1.171±0.117	0.0000
CDR2450	MerR-family transcriptional regulator	1.147±0.076	0.0000
CDR1950	putative transcriptional regulator	1.121±0.142	0.0000
CDR3274	GntR-family transcriptional regulator	1.106±0.108	0.0000
CDR0991	PadR-family transcriptional regulator	1.105±0.216	0.0000
CDR3067	MarR-family transcriptional regulator	1.083±0.095	0.0000
CDR1259	sigma-54 dependent regulatory protein	1.044±0.092	0.0000
CDR1504	GntR-family transcriptional regulator	1.026±0.267	0.0001
CDR2599	LysR-family transcriptional regulator	1.011±0.090	0.0000
CDR0506	TetR-family transcriptional regulator	-1.021±0.085	0.0000
CDR3197	GntR-family transcriptional regulator	-1.041±0.117	0.0000
CDR0745	GntR-family transcriptional regulator	-1.074±0.088	0.0000
CDR0817	GntR-family transcriptional regulator	-1.091±0.076	0.0000
CDR0373	sigma-54-dependent transcriptional regulator	-1.173±0.083	0.0000
CDR1311	GntR-family transcriptional regulator	-1.197±0.118	0.0000
CDR2781	GntR-family transcriptional regulator	-1.313±0.092	0.0000
CDR2975	AraC-family transcriptional regulator	-1.343±0.179	0.0000
CDR2929	*treR*; GntR-family transcriptional regulator	-1.635±0.142	0.0000
CDR2847	putative transcriptional regulator	-1.740±0.119	0.0000
Sigma Factors			
**Gene**	**Name, Description, & Details**	**L2FC±SE**	**p-adj**
CDR1348	*rpoD2*; RNA polymerase sigma factor RpoD	1.259±0.136	0.0000
CDR0050	*sigH*;RNA polymerase sigma-H factor	-1.259±0.066	0.0000
Transcription Antierminators			
**Gene**	**Name, Description, & Details**	**L2FC±SE**	**p-adj**
CDR2970	*bglG2*; transcription antiterminator	1.469±0.149	0.0000
CDR0690	putative transcription antiterminator	1.128±0.143	0.0000
CDR2222	*mtlR;* putative transcription antiterminator	1.036±0.087	0.0000
CDR0054	*nusG*; transcription antitermination protein	-1.098±0.075	0.0000
CDR2977	transcription antiterminator	-1.241±0.155	0.0000
Two Component Systems			
**Gene**	**Name, Description, & Details**	**L2FC±SE**	**p-adj**
CDR1476	putative two-component histidine kinase	1.557±0.098	0.0000
CDR1569	two-component sensor histidine kinase	1.260±0.141	0.0000
CDR1568	two-component response regulator	1.198±0.140	0.0000
CDR2187	two-component sensor histidine kinase	-1.393±0.079	0.0000
CDR0869	two-component system response regulator	-1.447±0.132	0.0000

CDR2188	two-component system response regulator	-1.679±0.108	0.0000
CDR2020	two-component sensor histidine kinase	-2.049±0.072	0.0000
CDR2021	two-component system response regulator	-2.372±0.089	0.0000
CDR2610	*hexK*; two-component sensor histidine kinase	-3.268±0.092	0.0000
CDR2611	*hexR;* two-component response regulator	-3.286±0.094	0.0000
CDR2206	two-component sensor histidine kinase	-4.054±0.119	0.0000
CDR2205	two-component response regulator	-5.025±0.122	0.0000
