# Supplementary material for: Clostridioides difficile-mucus interactions encompass shifts in gene expression, metabolism, and biofilm formation
Source: mSphere. 2024 Jun 5;9(6):e00081-24. doi: 10.1128/msphere.00081-24 (PMC11332178; doi:10.1128/msphere.00081-24)
Supplement: Text S1 — Supplemental materials and methods and Tables S3 and S4. [file msphere.00081-24-s0006.docx]

**Text S1.**

**SUPPLEMENTAL METHODS**

**Strain and plasmid construction.** Antibiotics were used as needed for selection or counterselection at the following concentrations: Chloramphenicol (Cm), 20 µg/ml; Ampicillin (Amp) 100 µg/ml; Thiamphenicol (Tm), 10 µg/ml; Kanamycin (Kan), 100 µg/ml; Anhydrotetracycline (Atc), 100 ng/ml. To make in-frame deletions in *C. difficile*, a previously established toxin-antitoxin allelic exchange plasmid, pMSR0, was used (1). Regions of homology (~1 kb) flanking the sequence to be deleted were amplified by PCR. Following digestion of pMSR0 with BamHI, homology arms were inserted into the plasmid using Gibson Assembly. After transformation into *E. coli* DH5α, Cm-resistant clones containing the insert were identified by PCR. Insert sequences were confirmed by Sanger sequencing. Plasmids were introduced into *C. difficile* by conjugation with *E. coli* HB101(pRK24) using heat shock as previously described (2). After 24-48 hours, transconjugants were selected on BHIS-TmKan agar, followed by counterselection on BHIS-Atc agar (3, 4). Deletion mutants were identified using PCR with primers flanking the homology region, followed by Sanger sequencing to confirm each deletion. Primers used in this study are in Table S4.

**Growth curves.** *C. difficile* cultures were grown approximately 12 hours in TY broth. Cultures were pelleted and washed at least once and resuspended in PBS to remove trace medium. *C. difficile* was added to minimal or defined media with or without 50 µg/mL mucus, or to 1X TY or 1X BHIS media, at a starting optical density at 600nm (OD_600_) of 0.05. Availability of IEC-derived mucus was limited, hence this relatively low concentration of mucus. OD_600_ measurements were taken up to 48 hours. Growth rates were determined by calculating the change in OD_600_ over time during exponential growth using the exponential growth equation in Prism 10, with the exponential growth phase defined by at least three consecutive OD_600_ measurements indicating linear growth. Serial dilutions of each culture were plated at the start of every curve and at exponential and/or stationary phase time points to enumerate CFU/mL and confirm growth.

**RNA isolation and RNA-Seq read processing.** Following RNA extraction with TriZol (5), aqueous layers containing nucleic acids were combined 1:1 with cold 70% ethanol and transferred to columns in a RNeasy kit (Qiagen) for wash and elution steps. On-column DnaseI treatment (Rnase-Free Dnase Set, Qiagen) was performed prior to elution. RNA was stored at -80°C prior to sequencing. After RNA-sequencing, read quality was assessed using FastQC (v0.11.8). Universal sequencing adapter sequences were removed using Trimmomatic (v0.36) (6). Because mucus was derived from human cell cultures, and because PhiX spike-ins are commonly used for quality control in high throughput sequencing workflows, we used Fastq_screen (v0.14.0) to remove any contaminating reads aligning to human mitochondria (Accession No. NC_012920.1) or PhiX (Accession No. NC_001422.1) (7). Trimmed and cleaned reads were aligned to the *C. difficile* R20291 genome (Accession No. FN545816.1) using non-gapping aligner Bowtie2 (v2.4.1) in global mode (8).

**Validation of RNA-Seq results by qRT-PCR**. *C. difficile* broth cultures in CDMM with or without mucus were prepared as described for growth curves. Samples were collected at exponential phase (OD600 ~0.5), and RNA was isolated as described for the RNA-Seq experiment. cDNA was synthesized using a high capacity cDNA reverse transcription kit with random hexamers (Applied Biosystems). cDNA synthesis reactions were also performed without reverse transcriptase to control for genomic DNA contamination. For all reactions, SensiMix SYBR & Fluorescein qPCR reagents (Meridian Biosciences) were used. Primers were used at a final concentration of 500 µM and 2 ng cDNA template were added per reaction. Primer sequences for qRT-PCR are in Table S4. qRT-PCR data were analyzed using ∆∆Ct method with *rpoC* as a housekeeping gene as previously described (4, 9, 10). Secondary normalizations were made to the no mucus condition.

**Biophysical and biochemical analysis of *ex vivo* mucus.** To determine the complex viscosity of *ex vivo* mucus, microscopic viscoelastic moduli were quantified using particle tracking microrheology (PTMR) as previously described (11). Fluorescent carboxylated beads (1 µm, ThermoFisher Scientific) were added at a 1:60 dilution at time of inoculation. At each time point, mucus (5 µL) was added to glass slides with a parafilm window and sealed with a glass coverslip. Brownian diffusion of the fluorescent beads was recorded using video microscopy, with motion tracked and quantified using custom Python and MATLAB scripts (TrackPy: <https://zenodo-org.libproxy.lib.unc.edu/records/7670439>). Viscoelastic moduli were calculated using particle mean squared displacement and the Stokes-Einstein relationship (12, 13). Multiangle laser light scattering (MALLS) was used to measure molecular weights, radii of gyration, and mucin concentrations in *ex vivo* mucus, as described(11). Mucus (10 µl) was diluted 1:100 in 6M guanidinium HCl (Fisher Scientific) to break noncovalent bonds, leaving large mucin molecules. Samples (300 µl) were injected into a 100 µL loop and eluted in light-scattering buffer containing 200 mM NaCl, 10 mM EDTA, and 0.01% NaN_3_. Samples were run through a size-exclusion chromatography column (Sepharose CL-2B, Cytiva) in series with MALS (DAWN Heleos II, Wyatt Technologies) and differential refractometry (Optilab, Wyatt Technologies). Mucin concentrations were recorded using the differential refractometer.

**Qualitative assessment of mucin composition.** To determine the mucin types produced by IECs, a direct collection of *ex vivo* mucus from the IEC model was prepared for label-free quantitative proteomics with filter-aided sample preparation (FASP) (14). The sample (100 µl) was denatured using 8M urea, followed by cysteine residue reduction with 10 mM dithiothreitol and alkylation using 50 mM iodoacetamide. The sample was then digested overnight at 37°C with pig trypsin (25 ng/µl). The resulting peptide mixture was vacuum freeze-dried, then dissolved in 30 µl of 1% acetonitrile and 0.1% trifluoroacetic acid, with 5 µl injected per technical replicate for LC-MS/MS analysis. A Q-Exactive mass spectrometer coupled with an Ultimate 3000 nano HPLC system was used for LC-MS/MS analysis, with data acquisitions following procedures outlined previously (15).

Proteomic data underwent processing and was searched against the UniProt protein database (Homo sapiens, November 2023) using Proteome Discoverer (v1.4). The Sequest search engine applied the following parameters: 10 ppm mass accuracy for parent ions, 0.02 Da accuracy for fragment ions, and allowance of 2 missed cleavages. Carbamidomethyl modification for cysteines was fixed, while methionine oxidation was considered variable. Scaffold (v5.3.0) was used for validating MS/MS-based peptide and protein identifications. Peptides were accepted with over 95.0% probability by the scaffold local FDR algorithm, while proteins required over 99.0% probability and a minimum of two identified peptides for acceptance. Protein probabilities were assigned using the Protein Prophet algorithm (16). Proteins containing similar peptides that could not be differentiated based on MS/MS analysis alone were grouped to satisfy the principles of parsimony. Protein intensities were calculated by adding the 3 highest peptide intensities per protein.

**Biofilm Assays.** Overnight cultures of *C. difficile* grown in TY broth were pelleted and washed with PBS, then diluted 1:30 in CDMM with or without 50 µg/mL mucus. Cultures were grown to late exponential phase (OD_600_ 0.8-1) then normalized to an OD_600_ of 0.5 before aliquoting into untreated 96-well polystyrene plates. After 24 hours, supernatants were removed, and biomass was fixed with 100% methanol for 20 minutes (17). After fixation, biofilms were stained with 0.1% w/v crystal violet for 30 minutes (18), after which they were gently rinsed with 1X PBS and resolubilized in 4:1 isopropanol acetone, similar to past work (19). To quantify biofilm, absorbance at 570 nm was measured.

**STRAIN AND PRIMER TABLES**

**Table S3**: Bacterial strains and plasmids used in this study.

| **Lab**  **Notation** | **Strain/Plasmid Name** | **Description** | **Reference** |
| --- | --- | --- | --- |
| AC472 | *E. coli* DH5ɑ | F- φ80lacZΔM15 Δ(lacZYA-argF)U169 *recA1 endA1* *hsdR17*(rk -, mk+) *phoA supE44 thi-1 gyrA96* *relA1* λ-*tonA* | Invitrogen (20) |
| RT273 | *C. difficile* R20291 | Ribotype 027 strain (Genbank Accession No. FN545816.1) | (21) |
| RT3118 | *C. difficile* R20291 ∆0453-0455 | Mutant with in-frame deletion of genes corresponding to locus tags CDR20291_0453-0455 | This work |
| RT3148 | *C. difficile* R20291 ∆0693-0696 | Mutant with in-frame deletion of genes corresponding to locus tags CDR20291_0693-0696 | This work |
| RT3120 | *C. difficile* R20291 ∆2495 | Mutant with in-frame deletion of genes corresponding to locus tags CDR20291_2495 | This work |
| RT270 | *E. coli* HB101(pRK24) | *E. coli* strain with plasmid for conjugations with *C. difficile*. Amp^R^, Cm^R^ | (22) |
| RT2460 | pMSR0 | Plasmid backbone containing toxin-antitoxin system for allelic exchange in *C. difficile*. Cm^R^ | (1) |
| RT3318 | pMSR0::0453-0455 | Allelic exchange plasmid containing homology arms flanking 0453-0455 region. Cm^R^ | This work |
| RT3320 | pMSR0::0693-0696 | Allelic exchange plasmid containing homology arms flanking 0693-0696 region. Cm^R^ | This work |
| RT3319 | pMSR0::2495 | Allelic exchange plasmid containing homology arms flanking 2495 region. Cm^R^ | This work |

**Table S4**: Primers used in this study.

| ***Cloning and Mutagenesis:*** | | |
| --- | --- | --- |
| **Lab Notation** | **Primer Name** | **Sequence (5’-3’)*** |
| R2743 | pMSR0 Ins Screen F | gtgttatcaattgcactactcatgg |
| R2744 | pMSR0 Ins Screen R | gttgaaccattagctaaggattcag |
| R3418 | 0453-0455 Screen F | ctttttcctgtgtcaatgc |
| R3419 | 0453-0455 Screen R | ctttttttgacagtatggcc |
| R3424 | 0453-0455 Gibson UP F | GATTTCTTTCAGTTTC**GGATCC**ggttttccatgtccagg |
| R3425 | 0453-0455 Gibson DN F | gacttataaagttttatcgtgtttttccatgatttcctcccca |
| R3426 | 0453-0455 Gibson UP R | tggggaggaaatcatggaaaaacacgataaaactttataagtc |
| R3427 | 0453-0455 Gibson DN R | GACGTCGACTCTAGA**ggatcc**caaacctatctgccaactc |
| R3651 | 0453-0455 Confirm Deletion F | cattaaacattttttcaccacc |
| R3652 | 0453-0455 Confirm Deletion R | gttttatccttaattaaggacatg |
| R3448 | 0693-0696 Screen F | gaggtaataatatggacccag |
| R3449 | 0693-0696 Screen R | gtagtgaaaatttgtcaggttc |
| R3454 | 0693-0696 Gibson UP F | GATTTCTTTCAGTTTC**GGATCC**gaagagtggcaataggc |
| R3455 | 0693-0696 Gibson DN F | gtgtatagaggaggataaattatatgactggtggacaagttatgtaag |
| R3456 | 0693-0696 Gibson UP R | cttacataacttgtccaccagtcatataatttatcctcctctatacac |
| R3457 | 0696-0696 Gibson DN R | GACGTCGACTCTAGA**ggatcc**gcatgatacagtagcaacg |
| R3549 | 0693-0696 Confirm Deletion F | gcagttagatatttgttagtggg |
| R3550 | 0693-0696 Confirm Deletion R | cttaattacaaaaatgaggctatctc |
| R3648 | 2495 Confirm pMSR0 Ins | cttcttcatccattgcacc |
| R3366 | 2495 Screen F | caagtaattctatagcattcgc |
| R3367 | 2495 Screen R | cagttcttgtcatatcagcac |
| R3372 | 2495 Gibson UP F | GATTTCTTTCAGTTTC**GGATCC**gctcttattattacgaatggag |
| R3373 | 2495 Gibson DN F | cattttatttagctgttttcttttccatcctaatttcatttccc |
| R3374 | 2495 Gibson UP R | gggaaatgaaattaggatggaaaagaaaacagctaaataaaatg |
| R3375 | 2495 Gibson DN R | GACGTCGACTCTAGA**ggatcc**ctttaagttgttccatagcc |
| R3521 | 2495 Confirm Deletion F | cagcatccttaattctctgtgc |
| R3522 | 2495 Confirm Deletion R | ctacttttgcattaaatacggaag |
| ***qRT-PCR:*** | | |
| R3318 | CDR0455 F | gcaattccaacaacttcaccag |
| R3319 | CDR0455 R | agcctctatagtactgttccctatg |
| R3612 | CDR0693 F | aaaggttctggaggatggg |
| R3613 | CDR0693 R | ttactgcagtttctggctttg |
| R3614 | CDR2495 F | cagcgcaaattaaagctcctg |
| R3615 | CDR2495 R | agctaagtctacagcatcaagc |
| R850 | rpoC F | ctagctgctcctatgtctcacatc |
| R851 | rpoC R | ccagtctctcctggatcaacta |

*****Restriction enzyme sites are bolded. Capital letters designate Gibson primer tails.

**REFERENCES:**

1. Girinathan BP, DiBenedetto N, Worley JN, Peltier J, Arrieta-Ortiz ML, Immanuel SRC, Lavin R, Delaney ML, Cummins CK, Hoffman M, Luo Y, Gonzalez-Escalona N, Allard M, Onderdonk AB, Gerber GK, Sonenshein AL, Baliga NS, Dupuy B, Bry L. 2021. *In vivo* commensal control of *Clostridioides difficile* virulence. Cell Host Microbe 29:1693-1708.e7.

2. Kirk JA, Fagan RP. 2016. Heat shock increases conjugation efficiency in *Clostridium difficile*. Anaerobe 42:1–5.

3. Girinathan BP, DiBenedetto N, Worley J, Peltier J, Lavin R, Delaney ML, Cummins C, Onderdonk AB, Gerber GK, Dupuy B, Sonenshein AL, Bry L. 2020. The mechanisms of in vivo commensal control of *Clostridioides difficile* virulence. bioRxiv https://doi.org/10.1101/2020.01.04.894915.

4. Reyes Ruiz LM, King KA, Agosto-Burgos C, Gamez IS, Gadda NC, Garrett EM, Tamayo R. 2022. Coordinated modulation of multiple processes through phase variation of a c-di-GMP phosphodiesterase in *Clostridioides difficile*. PLoS Pathog 18:e1010677.

5. Bouillaut L, McBride SM, Sorg JA. 2011. Genetic manipulation of *Clostridium difficile*. Curr Protoc Microbiol 20: 9A.2.1-9A.2.17.

6. Bolger AM, Lohse M, Usadel B. 2014. Trimmomatic: a flexible trimmer for Illumina sequence data. Bioinformatics 30:2114–2120.

7. Wingett SW, Andrews S. 2018. FastQ Screen: A tool for multi-genome mapping and quality control. F1000Res 7:1338.

8. Langmead B, Salzberg SL. 2012. Fast gapped-read alignment with Bowtie 2. Nat Methods 9:357–359.

9. Garrett EM, Sekulovic O, Wetzel D, Jones JB, Edwards AN, Vargas-Cuebas G, McBride SM, Tamayo R. 2019. Phase variation of a signal transduction system controls *Clostridioides difficile* colony morphology, motility, and virulence. PLoS Biol 17:1–28.

10. Trzilova D, Anjuwon-Foster BR, Torres Rivera D, Tamayo R. 2020. Rho factor mediates flagellum and toxin phase variation and impacts virulence in *Clostridioides difficile*. PLoS Pathog 16:1–28.

11. Howard RL, Markovetz M, Wang Y, Ehre C, Sheikh SZ, Allbritton NL, Hill DB. 2021. Biochemical and rheological analysis of human colonic culture mucus reveals similarity to gut mucus. Biophys J 120:5384–5394.

12. Mason TG. 2000. Estimating the viscoelastic moduli of complex fluids using the generalized Stokes-Einstein equation. Rheol Acta 39:371-378.

13. Hill DB, Vasquez PA, Mellnik J, McKinley SA, Vose A, Mu F, Henderson AG, Donaldson SH, Alexis NE, Boucher RC, Forest MG. 2014. A biophysical basis for mucus solids concentration as a candidate biomarker for airways disease. PLoS One 9:e87681.

14. Wiśniewski JR, Zougman A, Nagaraj N, Mann M. 2009. Universal sample preparation method for proteome analysis. Nat Methods 6:359–362.

15. Kesimer M, Cullen J, Cao R, Radicioni G, Mathews KG, Seiler G, Gookin JL. 2015. Excess secretion of gel-forming mucins and associated innate defense proteins with defective mucin un-packaging underpin gallbladder mucocele formation in dogs. PLoS One 10:e0138988.

16. Nesvizhskii AI, Keller A, Kolker E, Aebersold R. 2003. A statistical model for identifying proteins by tandem mass spectrometry. Anal Chem 75:4646–4658.

17. Vyas HKN, McArthur JD, Sanderson-Smith ML. 2021. An optimised GAS-pharyngeal cell biofilm model. Sci Rep 11:8200.

18. Purcell EB, McKee RW, Courson DS, Garrett EM, McBride SM, Cheney RE, Tamayo R. 2017. A nutrient-regulated cyclic diguanylate phosphodiesterase controls *Clostridium difficile* biofilm and toxin production during stationary phase. Infect Immun 85:e00347-17.

19. O’Toole GA, Pratt LA, Watnick PI, Newman DK, Weaver VB, Kolter R. 1999. Genetic approaches to study of biofilms. Methods Enzymol 310:91–109.

20. Hanahan D. 1983. Studies on transformation of *Escherichia coli* with plasmids. J Mol Biol 166:557–580.

21. Stabler RA, He M, Dawson L, Martin M, Valiente E, Corton C, Lawley TD, Sebaihia M, Quail MA, Rose G, Gerding DN, Gibert M, Popoff MR, Parkhill J, Dougan G, Wren BW. 2009. Comparative genome and phenotypic analysis of *Clostridium difficile* 027 strains provides insight into the evolution of a hypervirulent bacterium. Genome Biol 10:R102.

22. McBride SM, Sonenshein AL. 2011. Identification of a genetic locus responsible for antimicrobial peptide resistance in *Clostridium difficile*. Infect Immun 79:167–176.
